# Assessment of Dietary Intake, Energy Status, and Factors Associated With RED-S in Vocational Female Ballet Students

**DOI:** 10.3389/fnut.2018.00136

**Published:** 2019-01-09

**Authors:** Rita Civil, Antonia Lamb, Diana Loosmore, Louisa Ross, Kerry Livingstone, Fiona Strachan, James R. Dick, Emma J. Stevenson, Meghan A. Brown, Oliver C. Witard

**Affiliations:** ^1^Faculty of Health Science and Sport, University of Stirling, Stirling, United Kingdom; ^2^Modern Ballet, Royal Conservatoire of Scotland, Glasgow, United Kingdom; ^3^Institute of Aquaculture, University of Stirling, Stirling, United Kingdom; ^4^Institute of Cellular Medicine, Newcastle University, Newcastle upon Tyne, United Kingdom; ^5^School of Sport and Exercise, University of Gloucestershire, Cheltenham, United Kingdom

**Keywords:** energy availability, RED-S, menstrual dysfunctions, vitamin D, ballet dancers, bone mineral density (DXA), energy intake and expenditure, eating behaviors

## Abstract

Elite ballet dancers are at risk of health issues associated with Relative Energy Deficiency in Sport (RED-S). This study determined the nutritional status, estimated energy status, and assessed factors related to RED-S in vocational female ballet students. Using a cross-sectional study design, we measured dietary intake (food diaries and 24 h dietary-recall) and energy expenditure (accelerometry) in vocational female ballet students (*n* = 20; age: 18.1 ± 1.1 years; body mass index: 19.0 ± 1.6 kg·m^2^; body fat: 22.8 ± 3.4%) over 7 days, including 5 weekdays (with dance training) and 2 weekend days (without scheduled dance training). Furthermore, we assessed eating behaviors, menstrual function, risk of RED-S (questionnaires), and body composition (dual x-ray absorptiometry). Energy and macronutrient intakes of vocational ballet students were similar during weekdays and weekend days (*P* > 0.050), whereas total energy expenditure was greater on weekdays than weekend days (*P* < 0.010; 95% CI: 212, 379). Energy balance was lower on weekdays (−425 ± 465 kcal·day^−1^) than weekend days (−6 ± 506 kcal·day^−1^, *P* = 0.015; 95% CI: −748, −92). Exercise energy expenditure was greater on weekdays (393 ± 103 kcal·day^−1^) than weekend days (213 ± 129 kcal·day^−1^; *P* < 0.010; 95% CI: 114, 246), but energy availability was similar between time periods (weekdays 38 ± 13 kcal·kg FFM·day^−1^; weekend days 44 ± 13 kcal·kg FFM·day^−1^; *P* = 0.110). Overall, 35% of participants had an energy intake <1,800 kcal·day^−1^, 44% had reduced energy availability (30–45 kcal·kg FFM·day^−1^), and 22% had low energy availability (<30 kcal·kg FFM·day^−1^). Menstrual dysfunctions were reported in 40% of participants; 15 and 25% reported oligomenorrhea and secondary amenorrhea, respectively; while 65% were classified at risk of RED-S (based on the Low Energy Availability in Females Questionnaire). All participants had adequate bone health (bone mineral density Z-score: 1.1 ± 0.9 SD), but 20% had <85% expected body weight. The observation of an energy deficit in vocational female ballet students was primarily attributed to an inability to plan energy intake and thereby meet higher energy requirements during ballet training weekdays. Screening for factors associated with RED-S and tailoring education programs to inform energy and nutrition requirements for health and training are recommended in elite young ballet students.

## Introduction

Physique is integral to aesthetics and performance in elite ballet (1). In this regard, a lean and slim body physique is typically considered advantageous for artistic expression and producing powerful and graceful movements specific to ballet (2). However, the relentless desire to achieve the “ideal” body physique combined with other aspects such as high training demands and a competitive environment, place elite level ballet students and dancers at risk of a syndrome called Relative Energy Deficiency in Sport, or RED-S (3). This syndrome is characterized by impaired physiological function with negative health and performance consequences that extend beyond the initial Female Athlete Triad model (4). Such factors associated with RED-S include, but are not limited to, low bone mineral density (BMD), disrupted metabolic function [e.g., suppressed resting metabolic rate (RMR)], alterations in hormone levels, and impaired menstrual function (3). Accordingly, a previous study reported that 69% of professional ballet dancers experienced secondary amenorrhea (5). Moreover, a decreased RMR (6–9) and altered hormone levels (7, 10–12) are commonly reported in elite ballet dancers while some, but not all, studies have reported low BMD in female dancers (9, 13, 14). The consequences of RED-S may be more severe in young dancers such as vocational ballet students by affecting growth and development and limiting BMD that typically peak by the end of the second or early in the third decade of life (14).

The underlying physiological cause of RED-S is low energy availability (EA). A state of low EA results when dietary energy intake (EI) is insufficient to support the energy costs associated with exercise combined with all other physiological processes on-going in metabolically active tissues (15). Early laboratory studies identified a threshold level of <30 kcal·kg FFM·day^−1^ to represent low EA, below which physiological functions were impaired in healthy females (15). Although, an EA level of 30 kcal·kg FFM·day^−1^ was recognized to correspond with the value of RMR, more recent evidence suggests that this energy level may not provide a universal threshold across different body sizes during pubertal age (16). Accordingly, some individuals may experience physiological disruptions with reduced EA levels (30–45 kcal·kg FFM·day^−1^) (16). Optimal levels of EA for the healthy functioning of body systems and weight maintenance or energy balance (EB) are generally accepted to be ~45 kcal·kg FFM·day^−1^ (16). However, individuals that have previously experienced a period of low/reduced EA may currently have an adequate energy status and be weight stable, but symptoms of RED-S may still persist (16). Accordingly, several screening tools such as the Low Energy Availability in Females Questionnaire (LEAF-Q) have been developed to detect female athletes and dancers at risk of RED-S, based on self-reported physiological symptoms of low EA (17).

Low EA can exist both in the presence or absence of eating disorders/disordered eating and also can be caused by inadvertent under-eating (3). Previous research revealed low EA levels, a high prevalence of health-associated problems, and disordered eating behaviors in elite ballet dancers (18, 19). High training volumes with reduced opportunities to eat (20) or misguided weight loss practices (16) may contribute to low EA in dance populations. Hence, adequate energy intake and nutrition is critical for vocational ballet students to facilitate growth and development, while supporting the physiological demands of training (21).

A limited number of studies have simultaneously measured EI and total energy expenditure (TEE) to estimate energy status in elite dance populations (10, 22, 23). In this regard, we recently assessed EI and TEE, and calculated EB and EA in pre-professional female contemporary dancers during a typical 7-day period, including weekdays with scheduled dance training, and weekend days without scheduled dance training (22). Our findings revealed that dancers were in negative EB and low EA during weekdays, and in a marginal positive EB and reduced EA during weekend days (22). However, no study to date has simultaneously measured EI and TEE in pre-professional (i.e., vocational students) ballet dancers over a 7-day period, comparing weekdays and weekend days. Therefore, the primary aim of this study was to assess dietary intake and energy expenditure, and estimate EB and EA of vocational female ballet students during a typical 7-day period. A secondary study aim was to characterize the prevalence of factors associated with RED-S in this cohort of elite young ballet students.

## Materials and Methods

### Participants and Ethical Approval

Twenty students in full-time vocational ballet training attending the Royal Conservatoire of Scotland in Glasgow volunteered and completed the study. This sample size is similar to our prior study in pre-professional contemporary dancers (22), and a recent study that determined the prevalence of low EA indicators in elite female athletes (24). Participants were all Caucasian, young (18 ± 1 years of age), and were either enrolled in the 1st (*n* = 8), 2nd (*n* = 11), or 3rd (*n* = 4) years of study at the Royal Conservatoire. Exclusion criteria included any health condition that would prevent from successful participation in the study (i.e., epilepsy, bronchitis, severe asthma, cardiac complaints, bacterial or viral infection, pregnancy, disabling injury). Vocational ballet students were informed about the study design prior to signing an informed consent. The study was conducted during the autumn term of semester. Permission to undertake the study was provided by the Royal Conservatoire of Scotland and ethical approval was granted by The University of Stirling Research Ethics Committee and NHS East of Scotland Research Ethics Committee.

### Overview of Study Design

**Figure 1:**
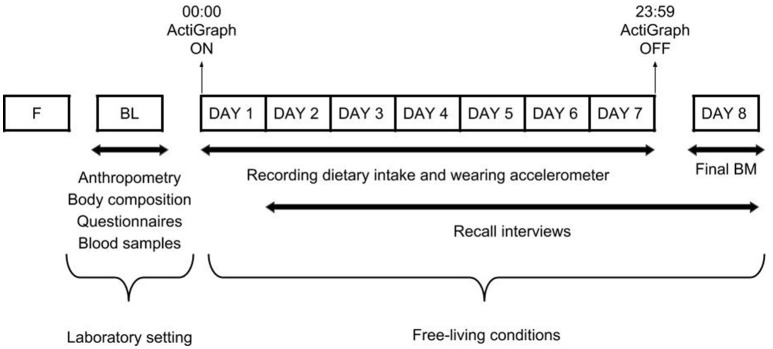
Overview of the study design. F, familiarization trial; BL, baseline measurements; BM, body mass.

In a cross-sectional design, and using similar methodology to our previous study in pre-professional contemporary dancers (22), our dataset was collected at baseline and throughout a 7-day period (Figure 1). At baseline, measurements of anthropometry, body composition, eating behaviors, menstrual function, risk of RED-S, and vitamin D status were collected. Thereafter, dietary intake was assessed using self-reported weighed food diaries and 24-h recall interviews. Energy expenditure was estimated using accelerometry and activity logs in the participants' normal environment. Energy intake and expenditure were assessed during 5 weekdays of scheduled dance training and 2 weekend days without scheduled dance training. All vocational female ballet students were encouraged to maintain their usual dietary and activity behaviors, including their scheduled dance training during this period.

### Body Composition

All anthropometric measurements were performed in the morning by the same trained researcher. Participants reported to the laboratory in a fasted (>10 h) and rested (i.e., avoiding intense exercise for at least 12 h) state, and emptied their bladders prior to measurements. Body mass (BM) was measured to the nearest 0.1 kg in minimal clothing using an electronic scale (Seca Quadra 808, Birmingham, UK). Height was measured without shoes and in minimal clothing using a stadiometer (Seca, Birmingham, UK) to the nearest 1 mm. Body mass index (BMI) was calculated and converted into percentiles using appropriate growth charts (25). Waist and hip circumference were measured to determine waist-to-hip ratio (22).

In accordance with the best practice protocol recommended by Nana et al. for measuring body composition (26), a Dual-Energy X-ray Absorptiometry (DXA) (Lunar iDXA GE Healthcare, USA) total body scan was conducted to determine body fat (BF), fat free mass (FFM), total body BMD and Z-scores. The typical technical error of measurement for iDXA machines using this protocol is ~1–2% (26). Participants were instructed to drink 500 ml of water within the 2 h before the scan in order to minimize potential errors in measurements caused by hypohydration (27). Scans were carried out using the automatic thickness mode chosen by the software and analyzed using GE Encore 13.40.038 Software (GE Healthcare, USA). The iDXA was calibrated daily with phantoms as per manufacturer guidelines. To ensure the accurate examination of BM changes over the 7-day period, a final measurement of BM was conducted under the same standardized conditions implemented at baseline. Baseline measurements of BM and FFM were used for all further estimations of RMR and EA, respectively.

### Self-Reported Eating Behaviors, Menstrual Function, and Energy Status

Participants completed the Healthier Dance Practice National Survey (28), which provided information regarding dance background, injury history, and general health status. A menstrual cycle questionnaire was completed to evaluate current and historical menstrual function. In addition, the 18 item Three Factor Eating Questionnaire (TFEQ) was used to assess eating behaviors of restrained eating (or conscious restriction of food intake in order to control body weight or to promote weight loss), uncontrolled eating (tendency to eat more than usual due to a loss of control over intake accompanied by subjective feelings of hunger), and emotional eating (inability to resist emotional cues) (29). Absolute scores were obtained for responses to all items coded on a four-point scoring scale. The degree of expression (0–100%) of each behavior was determined (30), with higher values indicating more expression of the behavior. The LEAF-Q (17) was administered to identify participants at risk of energy deficiency, and to complement the assessment of menstrual function. Eumenorrhea was defined as regular menstrual cycles (i.e., cycles occurring at intervals of 21–35 days). In terms of menstrual dysfunctions (MD), primary amenorrhea was defined as age at menarche of ≥15 years old, secondary amenorrhea was defined as the absence of ≥3 consecutive menses, and oligomenorrhea was defined as a cycle length >45 days (5).

### Dietary Intake

Dietary intake, including energy, macronutrient composition, fluid, and fiber intakes, was determined through the combined method of a self-reported weighed food diary and 24-h recall interviews, as previously described (22). This combined method has previously been shown to be a valid technique for quantifying food intake in male (31) and female (32) adolescent athletic populations. Prior to data collection, participants were familiarized with the format of food diaries, trained in appropriate recording methodology, and underreporting issues were discussed. Participants were then asked to complete a 7-day food diary, providing a detailed description of their food and fluid intake (i.e., quantity of item, brand names, timing of intake, cooking method). Where possible, photographic evidence was requested. Electronic portable scales (CS200 Ohaus Corp., USA) were provided to accurately record the weight of food items to the nearest gram. All measurements of dietary intake were conducted during free-living conditions and no attempts were made to influence the diet of participants.

Vocational female ballet students also were asked to take part in a 24-h diet recall interview using the two-pass method (33) for each day of the data collection period. The interview was carried out in person or over the phone when not possible to meet. The recall was cross-referenced with the food diary in order to add any missing data or clarify ambiguous information. Commercially available dietary analysis software (Nutritics Ltd, Ireland) was used to estimate EI and nutrient intake. The product label was consulted and nutrient composition was manually entered when foods or fluids were not listed in the database.

### Energy Expenditure

Individual RMR was estimated using the Harris-Benedict equation (34), as used previously (22). The thermic effect of food (TEF) was calculated for all macronutrients using values of 7.0, 27.5, and 2.5% for carbohydrate, protein, and fat, respectively (22). The thermic effect of activity was measured using a combination of accelerometry and data from activity logs. A tri-axial accelerometer (ActiGraph GT3X+, Pensacola, USA) was placed on the individuals' waist, and was secured with an elastic belt. The ActiGraph device was worn continuously throughout the 7-day study period, except during activities that would submerge the accelerometer in water (e.g., swimming, showering) or if removed for legitimate reasons (e.g., discomfort during sleep or when it was prohibited during performances or rehearsals) as per Brown et al. (22). Sixty-second sampling epochs were collected at a 60 Hz sample rate. Using data analysis software (ActiLife 5, ActiGraph), the Freedson VM3 combination algorithm (35) was used to estimate energy expenditure from the vector magnitude counts per minute of the three axes. In addition, participants wore a heart rate monitor strap (Polar H7, Kempele, Finland) during any active and training activities that connected to the ActiGraph by Bluetooth.

To account for missing data during periods when the accelerometer was removed, participants were required to register all non-wear periods (activity, time, and duration) on the activity log. Corrected Metabolic Equivalent (MET) values from the Compendium of Physical Activities (36) were used to estimate non-wear energy expenditure. An average 7-day MET value, as provided by the Actigraph device, was used in cases of battery failure or failure to record non-wear activity up to an 8-h period. Data from two participants were removed from the energy expenditure analysis due to technical difficulties with accelerometer equipment (i.e., two Actigraph batteries failed). Energy expenditure data collected during a weekend day from two participants also were excluded from the analysis due to non-wear periods without activity specification exceeding 8 h on that day. When using MET values, RMR was subtracted from each corresponding time period to ensure energy expenditure was not over-estimated.

### Estimating Energy Status

Daily TEE was calculated by combining RMR, TEF, and thermic effect of activity (combining data from the accelerometer and from non-wear periods) (37). Thermic effect of activity was separated into exercise energy expenditure (EEE) and non-exercise activity thermogenesis (NEAT) (37). In order to determine EEE, we defined the time periods that represented ‘exercise activity' by using the overall average active caloric expenditure (i.e., the average energy expenditure given as kcal·min^−1^ for all epochs with a heart rate value >79 beats·min^−1^ and vector magnitude count levels >1,951 counts·min^−1^) provided by Actigraph for each individual. Only calories expended during periods where the active caloric expenditure was greater or equal than the overall average were included in the calculation of EEE. Daily EB was estimated by subtracting TEE from EI (37). In order to address methodological limitations associated with variations in EI and TEE, EB was defined as values within 300 kcal of 0 since this energy value corresponds to the predicted amount of liver glycogen for female athletes (38). Daily EA was calculated by subtracting EEE from EI, and then dividing by FFM (37). The thresholds for defining a reduced EA and low EA were 30–45 kcal·kg FFM·day^−1^ and < 30 kcal·kg FFM·day^−1^, respectively.

### Vitamin D Status

Finger prick blood samples were collected in a fasted state for analysis of vitamin D concentrations. Two blood spots were dispensed onto specialized Whatman 903 blood collection cards (GE Healthcare Ltd, Cardiff) that were impregnated with 1% BHT in methanol. Once dried, samples were stored at −700°C until further analysis of 25-hydroxyvitamin D [25(OH)D] concentrations. Thereafter, blood spot samples were extracted using the DBS method of extraction (39) and analyzed by liquid chromatography tandem mass spectrometry (LC-MS/MS) utilizing components of the MassChrom® 25-OH-Vitamin D3/D2 in Serum/Plasma kit. In brief, a 6 mm circle was punched from the center of the dried blood spot and placed in a clean microtube (Eppendorf). 0.1% SDS solution was added, followed by internal standard mix (MassChrom® part no. 62004). The microtube was mixed before being placed in a shaking water bath at 40°C for 30 min. Acetonitrile was then added and the microtube returned to the water bath for a further 5 min. Following centrifugation at 10,000 rpm, the supernatant was transferred to a clean microtube before evaporation to dryness under nitrogen and re-suspension in methanol:water 70:30 for LC-MS/MS analysis. Samples were then run in duplicate in conjunction with the 6PLUS1 Multilevel Serum Calibrator Set (MassChrom® 25-OH-Vitamin D3/D2 on serum/plasma part no. 62039). 25(OH)D concentrations were converted for haematocrit level (39).

### Data Presentation and Statistical Analysis

Data are presented as means ± standard deviations (SD), unless otherwise specified. All data were normally distributed as determined by the Shapiro–Wilk test. Student's paired samples *t*-tests were used to analyse differences in BM measured at baseline and the final time-point. The daily average of TEE (kcal), EEE (kcal), EI (kcal), EA (kcal·kg FFM·day^−1^), macronutrient (% of EI, g and g·kg^−1^), fiber (g), fluid (ml), and alcohol (g) intakes were determined for three periods: the total 7-day recording period, weekdays days (average of 5 days), and weekend days (average of 2 days). Paired *t*-tests were used to compare EI, macronutrient, fiber, and fluid intakes, TEE TEF, EEE, NEAT, EB, and EA between weekday and weekend periods. A Pearson correlation coefficient was used to determine the relationship between menstrual function/LEAF-Q scores and EA levels. Correlation values (*R*^2^) were set as <0.2: weak correlation, 0.5: medium correlation, and >0.8: strong correlation. Significance level was set at *P* < 0.05, and 95% confidence intervals (95% CI) presented for significant differences. Data analysis was conducted using SPSS v19 (SPSS Inc., Chicago, USA).

## Results

### Participant Characteristics

Table 1 summarizes participant characteristics for anthropometry and body composition, health parameters, and training history in vocational female ballet students. Participants lost 0.47 ± 0.64 kg BM during the 7-day period (*P* = 0.004; 95% CI: 0.16, 0.76). Body composition measurements of BF%, FFM and BMD were within the normal range. The LEAF-Q score was ≥8, which is above a normal/healthy score value. Eleven participants (55%) had 25(OH)D concentrations ≥50 nmol·L^−1^, which is above vitamin D deficiency levels.

**Table 1:** Participant characteristics and health parameters.

	***n* = 20, mean ± SD**
Age (y)	18.1 ± 1.1
BM baseline (kg)	54.4 ± 5.8
BM final (kg)	54.0 ± 5.7*
Height (m)	1.69 ± 0.54
BMI (kg·m^2^)	19.0 ± 1.6
W:H	0.73 ± 0.19
BF (%)	22.9 ± 3.4
FFM (kg)	42.5 ± 4.0
Total BMD (g·cm^2^)	1.176 ± 0.771
Z-score (SD)	1.145 ± 0.930
25(OH)D (nmol·L^−1^)	60.8 ± 27.9
LEAF-Q score	8.8 ± 4.4
Self-reported training volume (h·week^−1^)	30.9 ± 6.1

*BM, body mass; BMI, body mass index; W:H, waist to hip ratio; BF, body fat measured by dual-Energy X-ray Absorptiometry; FFM, fat free mass measured by Dual-Energy X-ray Absorptiometry; BMD, bone mineral density; 25(OH)D, 25-hydroxyvitamin D; LEAF-Q, Low Energy Availability in Females Questionnaire*.

* *Denotes significant difference vs. baseline measurement (P < 0.050)*.

### Eating Behaviors

**Figure 2:**
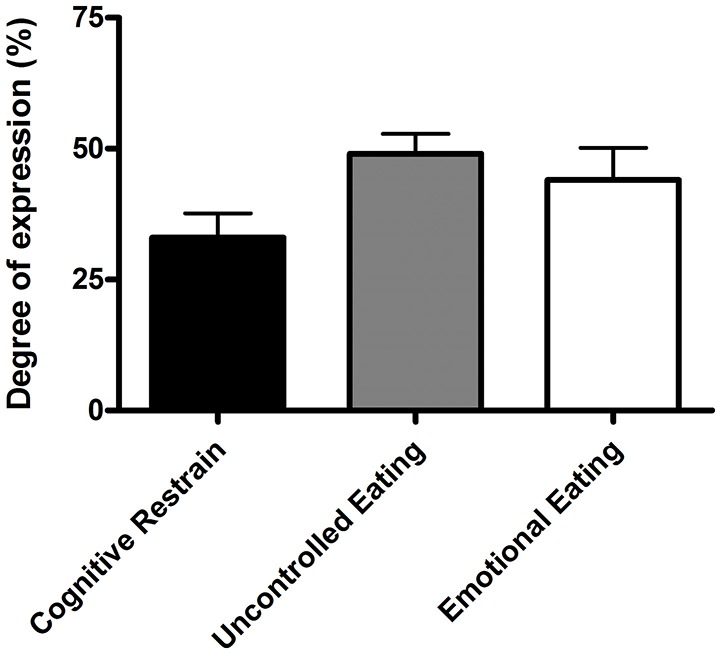
Eating behaviors of cognitive restrain, uncontrolled eating, and emotional eating. Values are means ± standard error of mean (*n* = 20).

Based on values calculated from the TFEQ, the most prevalent eating behavior was uncontrolled eating (Figure 2). In total, seven of 20 participants (35%) self-reported following a restricted diet with 1 vegan, 3 vegetarians, 2 non-dairy, and 1 weight-reducing.

### Estimated Energy Status

**Figure 3:**
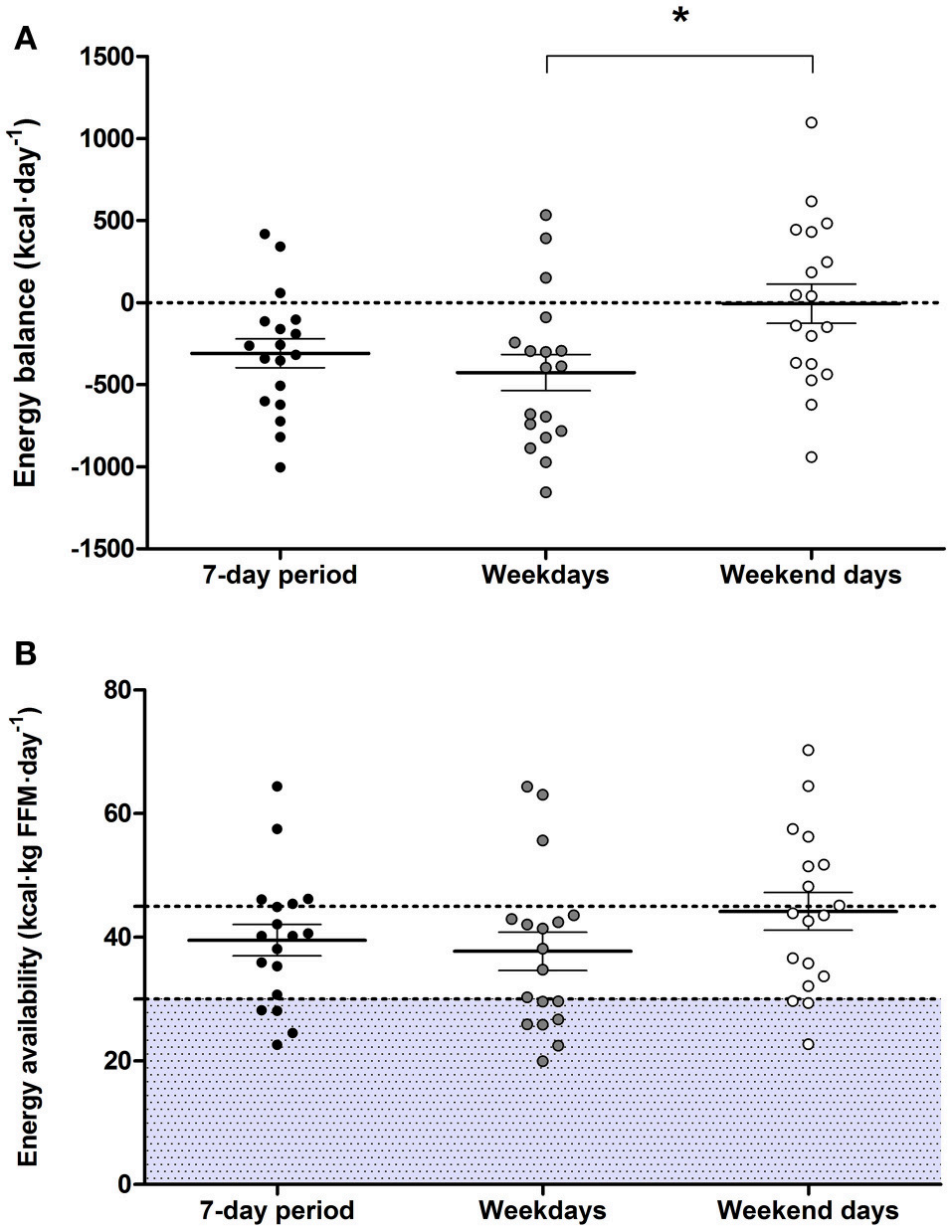
Energy balance **(A)** and energy availability **(B)** over the 7-day period, weekdays, and weekend days. Two dancers were removed from the this data analysis due to technical difficulties with accelerometer equipment, and a weekend day was excluded from the analysis from two different participants due to non-wear periods without activity specification >8 h. Values are means ± standard error of mean (*n* = 18), *denotes significant difference (*P* < 0.050). Shaded area indicates levels of low energy availability (< 30 kcal·kg FFM·day^−1^).

Vocational female ballet students had an EI lower than TEE when assessed for the overall 7-day period (*P* = 0.003; 95% CI: −493, −123) and on weekdays (*P* = 0.001; 95% CI: −657, −194), but not during weekend days (*P* = 0.958; Table 2. Values of TEE (95% CI: 212, 379), NEAT (95% CI: 90, 158), and EEE (95% CI: 114, 246) values were higher during weekdays than weekend days (*P* < 0.010; Table 2). A negative EB (−308 ± 372 kcal·day^−1^) was observed during weekdays, whereas an EB (−6 ± 506 kcal·day^−1^) was achieved on weekend days (*P* = 0.015; 95% CI: −748, −92) (Figure 3A). There were no statistical differences in EA between weekdays than weekend days (*P* = 0.110; Figure 3B). Over the 7-day period, 50% of participants (*n* = 9) had an energy deficit >300 kcal·day^−1^, 44% (*n* = 8) had reduced EA (30–45 kcal·kg FFM·day^−1^), and 22% (*n* = 4) had low EA (<30 kcal·kg FFM·day^−1^).

**Table 2:** Energy status of vocational female ballet students for the entire 7-day period, weekdays, and weekend days.

	**7-day period**	**Weekdays**	**Weekend days**	***P* value**
EI (kcal·day^−1^)	2013 ± 398	1978 ± 455	2102 ± 606	0.423
TEE (kcal·day^−1^)	2319 ± 221	2403 ± 224	2108 ± 262	0.000*
RMR (kcal·day^−1^)	1408 ± 61	1408 ± 61	1408 ± 61	–
TEF (kcal·day^−1^)	165 ± 30	164 ± 35	168 ± 41	0.728
NEAT (kcal·day^−1^)	403 ± 107	436 ± 111	312 ± 114	0.000*
EEE (kcal·day^−1^)	344 ± 98	393 ± 103	213 ± 129	0.000*
EB (kcal·day^−1^)	−308 ± 372	−425 ± 466	−6 ± 506	0.015*
EA (kcal·kg FFM·day^−1^)	39.5 ± 10.8	37.7 ± 13.1	44.2 ± 12.9	0.110

*EI, energy intake; TEE, total energy expenditure; RMR, resting metabolic rate; TEF, thermic effect of food; NEAT, non-exercise activity thermogenesis; EEE, exercise energy expenditure. Values are means ± SD (n = 18). P-values refer to the comparison between weekdays and weekend days*,

* *denotes significant difference (P < 0.050) between weekdays and weekend days*.

### Energy, Macronutrient, Fiber, and Fluid Intakes

Energy and carbohydrate, protein, and fat intakes, expressed in absolute terms (g·day^−1^), relative to BM (g·kg·day^−1^) and as a % of EI, were similar on weekdays and weekend days (*P* > 0.050; Table 3). However, vocational ballet students consumed less fiber (*P* = 0.002; 95% CI: 2.4, 9.1) and fluids (*P* < 0.001; 95% CI: 276, 559) during weekend days (Table 3). In total, 4 participants consumed alcohol over the 7-day period (8.2 ± 21.4 g·day^−1^), and alcohol consumption was similar between weekend days and weekdays (*P* = 0.204). Regular supplement use was common among our ballet students (*n* = 11, 55%) and included multivitamins, vitamins C, D, and B12, antioxidants, probiotics, herbal extracts, minerals (zinc, iron, calcium, magnesium, manganese, potassium, selenium, iodine, cooper, chloride), cod liver oil, and fish oil.

**Table 3:** Dietary intake of pre-professional ballet dancers on weekdays, weekend days, and for the entire 7-day period.

	**7-day period**	**Weekdays**	**Weekend days**	***P*-value**
Energy intake (kcal·day^−1^)	2, 002 ± 415	1, 979 ± 470	2, 058 ± 589	0.594
Energy intake (kcal·kg·day^−1^)	37.0 ± 8.0	36.7 ± 9.4	37.8 ± 10.3	0.679
Carbohydrate (g·day^−1^)	262.8 ± 54.6	263.4 ± 66.4	261.2 ± 63.6	0.904
Carbohydrate (g·kg^−1^·day^−1^)	4.9 ± 1.1	4.9 ± 1.3	4.8 ± 1.1	0.804
Carbohydrate (%)	53.4 ± 4.0	53.6 ± 4.2	52.8 ± 6.4	0.595
Protein (g·day^−1^)	65.8 ± 12.3	65.5 ± 15.1	66.4 ± 17.6	0.857
Protein (g·kg^−1^·day^−1^)	1.2 ± 0.2	1.2 ± 0.3	1.2 ± 0.3	0.841
Protein (%)	13.9 ± 2.6	14.1 ± 3.1	13.5 ± 3.2	0.527
Fat (g·day^−1^)	74.7 ± 21.8	73.4 ± 22.5	77.9 ± 32.0	0.501
Fat (g·kg^−1^·day^−1^)	1.4 ± 0.4	1.4 ± 0.4	1.4 ± 0.6	0.563
Fat (%)	32.7 ± 5.0	32.6 ± 4.8	33.0 ± 6.9	0.791
Fiber (g·day^−1^)	30.1 ± 12.1	31.8 ± 13.1	26.0 ± 11.0	0.002*
Fluids (ml·day^−1^)	1, 649 ± 488	1, 768 ± 521	1, 350 ± 472	0.000*

*Values are means ± SD (n = 20). P-values refer to the comparison between weekdays and weekend days*,

* *denotes significant difference (P < 0.050) between weekdays and weekend days*.

### Factors Associated With RED-S

Table 4 displays the prevalence of factors associated with RED-S in all 20 vocational ballet students combined, and also divided into six categories depending on whether participants cumulatively reported 0, 1, 2, 3, 4, or 5 of these factors. Menstrual dysfunctions included secondary amenorrhea, reported in five participants (25%); and oligomenorrhea, reported in three (15%) participants. There were no associations between menstrual dysfunctions and EA levels (*R*^2^ = 0.342; *P* = 0.165; *n* = 18) or LEAF-Q scores and EA levels (*R*^2^ = 0.390; *P* = 0.109; *n* = 18) in our cohort of vocational female ballet students.

**Table 4:** Prevalence of factors associated with RED-S in vocational female ballet students.

	**Total**	**0 Factors**	**1 Factor**	**2 Factors**	**3 Factors**	**4 Factors**	**5 Factors**
Number of participants	20 (100%)	4 (20%)	6 (30%)	6 (30%)	2 (10%)	2 (10%)	0 (0%)
Menstrual dysfunctions	8 (40%)	0	0	4	2	2	0
LEAF-Q score ≥8	13 (65%)	0	3	6	2	2	0
BMD Z-score ≤−1	0 (0%)	0	0	0	0	0	0
<85% expected body weight	4 (20%)	0	2	0	0	2	0
EI <1,800 kcal·day^−1^	7 (35%)	0	1	2	2	2	0

*LEAF-Q, Low Energy Availability in Females Questionnaire; BMD, bone mineral density; EI, energy intake*.

## Discussion

This descriptive study simultaneously assessed the habitual dietary intake, energy expenditure, and subsequent energy status of vocational female ballet students during a typical 7-day period, including five dance training weekdays and 2 weekend days without scheduled dance training. Consistent with previous findings in young elite ballet students (8, 40), we estimated an energy deficit of ~308 kcal·day^−1^ over the total 7 days. This energy deficit was more pronounced during dance training weekdays since, overall, vocational ballet students maintained levels of EB during weekend days when not undertaking scheduled dance training. Over the 7-day period, EA was ~40 kcal·kg FFM·day^−1^, with reduced (30–45 kcal·kg FFM·day^−1^) and low (< 30 kcal·kg FFM·day^−1^) EA levels recorded in 44 and 22% of participants, respectively. We also report a high prevalence of menstrual dysfunctions (40%) and risk of RED-S, with 65% of vocational ballet students recording LEAF-Q scores ≥8.

Our recent study in pre-professional female contemporary dancers directly compared both EI and TEE between weekdays with scheduled dance training, and weekend days without scheduled dance training (22). Consistent with these findings (22), in the present study of vocational ballet students we report a negative EB on weekdays compared to EB levels on weekend days. Unlike our previous study (22), EA levels remained similar between weekdays and weekend days and we report similar EI and macronutrient intakes between weekdays and weekend days. Therefore, the difference in EB was primarily due to a greater energy expenditure (i.e., NEAT and EEE) during weekdays in the present study. Previous studies in elite ballet dancers report training volumes of 20–48 h per week (8–10). Our vocational ballet students self-reported a training volume of ~30 h per week, including periods of lower intensity activity (e.g., warm-up, stretching, barre phase) and resting periods (41). This activity pattern explains the moderate TEE of 2,319 ± 221 kcal·day^−1^ recorded in our study cohort. Defined as the energy cost exclusively from exercise, EEE is used to calculate EA. We rigorously determined EEE using a combined approach of heart rate monitors and accelerometry to determine an active energy expenditure threshold for each individual while adjusting for low intensity or sedentary activities. This adjustment is prudent for individuals undertaking high volumes of training with a moderate energy cost in order to avoid overestimation of EEE and underestimation of EA (16). Despite the moderate TEE and EEE recorded for our vocational ballet students, their EI was insufficient to support the higher energy requirements of training. This observation implies that elite ballet students would benefit from a better understanding of their training energy needs and nutrition strategies required for optimal health and performance.

The 7-day EI of our vocational ballet students was ~2,000 kcal per day, and is consistent with previous findings in young (39, 40, 42) and elite (23, 43) ballet students. In contrast, studies in senior professional (8) and pre-professional (10, 44) ballet dancers reported a lower dietary EI (~1,600 kcal per day), and one study reported higher (~2,400 kcal per day) values for daily EI (45). These variable findings may be due to differences in age and experience level (e.g., pre-professional vs. professional) of ballet dancers, as well as methodological limitations related to recording EI (37). In this regard, ballet dancers previously under-reported EI by 21% when using self-reported food diaries compared to EI values calculated using doubly labeled water methodology (46). Nonetheless, the combined method of weighed food diaries and dietary recall interview techniques, as utilized in this study, is known to minimize errors of measurement related to participant compliance and motivation and has previously been demonstrated to result in good agreement with actual dietary intake (31, 32). Using this combined approach, we reveal that our vocational ballet students recorded an EI insufficient to meet their energy requirements during training. Moreover, 35% of participants reported an EI of <1,800 kcal·day^−1^. Consistent with this notion, it has been proposed that an EI <1,800 kcal·day^−1^ is insufficient for females athletes during intense training to achieve adequate nutrition and energy for optimal health and performance (47).

The likelihood of an individual exhibiting a nutrient deficiency is increased with a low EI. To our knowledge, no consensus guidelines exist regarding recommended nutrient intakes for dance populations, although general nutritional recommendations have been published (48). In the present study, dietary carbohydrate and protein intakes were consistent with recommendations for skill-based adolescent athletes (21). With regards to dietary protein guidelines, protein intakes ~1.8 g·kg·day^−1^ divided evenly between meal servings are increasingly recommended during periods of energy restriction with the basis to offset the loss of muscle mass during weight loss (49). Hence, it could be argued that our ballet students successfully achieved their protein requirement, but not necessarily context-specific protein recommendations. In contrast, and consistent with previous findings in dance students (10, 22, 42), participants in the present study met dietary fat and fiber recommendations of <30% of total EI and 25-35 g·day^−1^, respectively (6). We acknowledge that our dietary assessments did not include micronutrient intakes. However, we did measure blood 25(OH)D concentrations as a biomarker of vitamin D status which also is influenced by sunlight exposure. This analysis revealed a blood 25(OH)D concentration of ~60 nmol·L^−1^ in our ballet students which is lower than previous values reported in young ballet students from New Zealand (~75 nmol·L^−1^) (42). While 55% of participants met the vitamin D recommendation of ≥50 nmol·L^−1^ (21) in the month of November, the northern latitude and indoor training characteristic of our cohort may place our cohort at risk of vitamin D insufficiency/deficiency, during both summer and winter months, due to reduced sun exposure (50). Although, no other clinical marker of nutrient status was assessed, we found that micronutrient supplement use was common practice among our vocational ballet students.

The desire to reduce BM or fat mass can lead to unhealthy or misguided dietary restrain in elite environments (3). Nevertheless, based on eating behavior results from the TFEQ (29), the motivation behind the insufficient EI in our vocational ballet students was unclear. Consistent with previous observations in teenagers and young adults (51), our participants recorded a degree of expression for cognitive restrain (33%) and emotional eating (44%). In contrast, our vocational ballet students recorded markedly higher values for behaviors of uncontrolled eating (49%) compared to values recorded by the control group in the same previous study (35%) (48). These observations suggest that our participants were often inadvertently under-eating, and compensated for a decreased EI by uncontrolled episodes of eating. Previous research highlights an increased risk of disordered eating among dancers (8, 18, 19) and athletes (2), when body physique is important for performance and appearance. Regardless of the reason behind a decreased EI, individuals may not be aware of the detrimental consequences of such behaviors and would benefit from early interaction with a professional (e.g., sports dietitian) that can enhance dietary practices and advice on how to achieve body physique goals in a safe and sustainable manner (16).

A mismatch between EI and EEE can occur both in the presence and absence of disordered eating/eating disorders and can lead to low EA (<30 kcal·kg FFM·day^−1^) (3). EA levels of our vocational female ballet students was ~40 kcal·kg FFM·day^−1^. Although this EA level represents a reduced EA (30–45 kcal·kg FFM·day^−1^) (16), it exceeds values previously recorded in pre-professional contemporary (21) and ballet (10) dancers, as well as professional ballet dancers (8, 19). The discrepancy in EA levels between studies may be due, at least in part, to methodological differences and limitations in estimating EA (16). In fact, recent work by Burke et al. (16) highlights the pitfalls of estimating EA and the lack of clear guidelines for the calculation of EA in free-living conditions, including the methods used to measure each components of the EA equation (i.e., EI, EEE, and FFM). With due consideration of these challenges, we estimated EA using rigorous methodology to determine each of these components. However, we acknowledge that a snapshot estimation of EA may not provide an accurate assessment of the health and true energy status of the individual (16). In this regard, field studies have failed to detect associations between estimated EA and objective measures of disrupted metabolic hormones and MD (16). In the present study, we failed to observe associations between EA and self-reported menstrual dysfunctions and physiological symptoms of low EA assessed by the LEAF-Q.

Menstrual irregularities are a common symptom of RED-S, and low EA is considered the primary cause of functional hypothalamic amenorrhea (3). In the present study, menstrual dysfunctions were prevalent in vocational female ballet students, whereby 40% of participants self-reported MD, where 25% reported secondary amenorrhea and 15% reported oligomenohrrea. Previous research reported high prevalence rates (20–40%) of secondary amenorrhea in professional ballet dancers, compared to a lower prevalence (0–4%) in controls (7, 8). We conceivably underestimated the prevalence of MD since subclinical MD were not assessed and hormonal contraceptives may have masked the potential presence of MD in five participants. Moreover, young females with a gynecological age <14 years may have increased sensitivity to low EA effects on luteinising hormone pulsatility, leading to MD (52). However, in this study vocational female ballet students self-reported MD, thereby the true cause of these disturbances in our cohort is not clear. The LEAF-Q (17) has previously been recommended to identify females at risk of low EA or RED-S (16) and is a validated screening tool whereby individuals self-report physiological symptoms of low EA (17). According to LEAF-Q results, we identified that 65% of our participants were at risk of RED-S with a score ≥8. This prevalence rate is higher than recently reported in professional ballet dancers (9), whereby 40% of dancers were at risk.

Despite the fact that BMI is not a true measure of body composition, it is often used as an indicator of chronic low EA (3). Using BMI-for-age growth charts, 20% of participants in our cohort were below the 5th percentile and were classified as underweight (<85% of expected body weight). In comparison, a recent study classified 50% of professional ballet dancers as underweight (BMI < 18.5 kg·m^2^) (9). These combined observations are not consistent with previous findings by Wyon et al. (1) that reported a higher BMI in professional ballet dancers compared to young elite ballet students (1). Bone health, including BMD, bone microarchitecture, and bone turnover markers also are known to be impaired with low EA (3). Nevertheless, the overall BMD (Z-score 1.145 ± 0.930 SD) among our vocational female ballet students, consistent with previous studies in professional ballet dancers (9, 13), was within normal range, and none of our participants reported low BMD (Z-score ≤ −1). These data suggest that weight bearing ballet-specific training may be protective for bone health, despite the presence of MD and the negative effect of low EA (14). Importantly, optimal bone mineral accrual during adolescence is key to ensure an adequate peak bone mass and improve bone health later in life (21). In the present study, overall, 80% of vocational ballet students reported ≥1 factors related to RED-S, where 60% may be slightly at risk (1 or 2 factors) and 20% at higher risk (3–4 factors). Therefore, our data support the relevance of monitoring symptoms of RED-S when screening for low EA in at-risk populations.

Due to metabolic adaptations, weight stability and EB can occur in cases of low EA (16). However, since we did not directly measure RMR, it is unclear whether our vocational ballet students experienced impaired metabolic function. Overall, we observed an ~0.5 kg decline in BM over the study period, which is consistent with the observation of a negative EB (~308 kcal·day^−1^). We acknowledge that this reduction in BM is significant given the low BM of our participants (~54 kg), however contend that this energy deficit and the subsequent BM reduction may not be sustained long-term. In this regard, follow up studies that measure dietary intake and energy expenditure over prolonged (e.g., 4–6 weeks) time periods are warranted in dancers. On the other hand, energy needs for growth also increase the energy demands of young individuals (21). Furthermore, dietary patterns observed in our ballet students such as a high intake of fiber, caffeine, and low energy density food/drinks, and poor energy distribution throughout the day may exacerbate the negative effects of low EA (3). Specifically, within-day periods with energy deficits >300 kcal^−1^ are associated with metabolic and endocrine alterations in female athletes, including MD (53). In the present study, although we did not estimate within-day EB, 9 of 18 participants reported an overall energy deficit >300 kcal·day^−1^ and patterns of skipping meals were anecdotally observed among some participants.

This descriptive study in vocational ballet students has several strengths, including the novel and rigorous method of combining self-reported weighed food diaries and 24-h dietary recall interviews to assess dietary intake in parallel with the measurement of energy expenditure over a 7-day period. However, several limitations must be highlighted with regards to these two measurements. First, field techniques such as food records and accelerometry may both underestimate and overestimate true values of dietary intakes and energy expenditure (37), and a snapshot assessment may not accurately represent an individual's long-term energy status (16). Moreover, we estimated RMR using Harris-Benedict equation, which has previously been reported to better estimate RMR in ballet dancers compared to controls (8). Second, recent findings in professional ballet dancers suggest the Cunningham equation is more suitable to detect female dancers with impaired metabolic function (9). Therefore, it is possible that we overestimated RMR for participants with MD and/or chronic metabolic adaptations. Finally, menstrual cycle phase on entering the study differed among participants, which may have affected individual EI and TEE, and BM (54). Therefore, including measurements of reproductive hormones and RMR would have helped identifying menstrual phase and status, and metabolic adaptations in our vocational female ballet students, respectively.

## Conclusions

In the present study, the failure of vocational female ballet students to meet their estimated energy requirements was due to an inability to plan weekly energy intakes to meet higher energy requirements during training weekdays. Our findings also suggest that vocational female ballet students may be at risk of RED-S, with reduced EA (30–45 kcal·kg FFM·day^−1^) and high LEAF-Q scores. Taken together, these data support the recommendation to screen elite young ballet students for factors associated with RED-S and tailor education programs to inform energy and nutrition requirements for health and training in this “at-risk” population.

## Author Contributions

The study was designed by RC, DL, LR, KL, MB, and OW. Data were collected by RC and AL. Analyzed by RC, AL, FS, and JD. Data interpretation and manuscript preparation were undertaken by RC, ES, MB, and OW.

### Conflict of Interest Statement

The authors declare that the research was conducted in the absence of any commercial or financial relationships that could be construed as a potential conflict of interest.
